# Gene expression analysis of nidus of cerebral arteriovenous malformations reveals vascular structures with deficient differentiation and maturation

**DOI:** 10.1371/journal.pone.0198617

**Published:** 2018-06-13

**Authors:** Jaya Mary Thomas, Sumi Surendran, Mathew Abraham, Dhakshmi Sasankan, Sridutt Bhaadri, Arumugam Rajavelu, Chandrasekharan C. Kartha

**Affiliations:** 1 Cardio Vascular Diseases and Diabetes Biology Program, Rajiv Gandhi Centre for Biotechnology, Poojapura, Thycaud, Thiruvananthapuram, Kerala, India; 2 Manipal Academy of Higher Education, Manipal, Karnataka, India; 3 Department of Neurosurgery, Sree Chitra Tirunal Institute for Medical Sciences & Technology, Thiruvananthapuram, Kerala, India; 4 Tropical Disease Biology Program, Rajiv Gandhi Centre for Biotechnology, Poojapura, Thycaud, Thiruvananthapuram, Kerala, India; Universität Stuttgart, GERMANY

## Abstract

**Objective:**

Arteriovenous malformations (AVMs) are characterised by tangles of dysplastic blood vessels which shunt blood from arteries to veins with no intervening capillary bed. It is not known at what stage of development and differentiation, AVM vessels became aberrant. To address this, we have analysed the expression of vascular differentiation, vascular maturation and brain capillary specific genes in AVM nidus.

**Methodology:**

We performed immunohistochemistry and western blot analysis of vascular differentiation (*HEY2*, *DLL4*, *EFNB2*, and *COUP-TFII*), vascular maturation (*ENG* and *KLF2*) and brain capillary specific genes (*GGTP* and *GLUT1*) on ten surgically excised human brain AVMs and ten normal human brain tissues.

**Results:**

Immunohistochemical analysis revealed that AVM vessels co-express both artery and vein differentiation genes. H-score analysis revealed that there is statistically significant (P < 0.0001) increase in expression of these proteins in AVM vessels compared to control vessels. These findings were further confirmed by western blot analysis and found to be statistically significant (P < 0.0001 and P < 0.001) for all proteins except Hey2. Both immunostaining and western blot analysis revealed that AVM vessels express GGTP and GLUT1, markers specific to brain capillaries. Immunofluorescent staining demonstrated that expression of KLF2, a vascular maturation marker is significantly (P <0.001) decreased in AVM vessels and was further confirmed by western blot analysis (P < 0.001). Immunohistochemical and western blot analysis demonstrated that another vascular maturation protein Endoglin had high expression in AVM vessels compared to control vessels. The results were found to be statistically significant (P < 0.0001).

**Summary:**

Our findings suggest that vascular structures of AVMs co-express markers specific for arteries, veins and capillaries. We conclude that AVM nidus constitutes of aberrant vessels which are not terminally differentiated and inadequately matured.

## Introduction

Arteriovenous malformation (AVM) of the brain is a devastating vascular malformation characterized by direct connections of dilated arteries and veins and a dearth of the normal capillary system. Feeding artery, draining vein and nidus are the three parts of the AVM lesion. These lesions can occur in any of the four lobes of the brain and patients with AVM present symptoms such as head ache, seizures, hemorrhage etc. Based on its location, size of lesion and type of venous drainage, surgical or nonsurgical treatment strategies are selected for AVM cure [1].

Despite much understanding on normal vascular development, mechanisms in the formation of discrete AVM lesions are unclear. Reports on the *de-novo* formation of AVM have challenged the view on its congenital origin [2]. Previous studies on AVM suggest that multiple pathways rather than a single pathway are involved in AVM pathogenesis [3]. Vascular malformations could occur when there is a defect in either early or late phase of vascular development.

Various hierarchical signaling molecules are involved in the early and late stages of vascular development. The initial stage of vascular development consists of the formation of a primitive vascular tube made of a single layer of endothelial cells. Predetermined genetic factors expressed in the endothelial cells decide the fate of the tube to develop as an artery or a vein [4, 5]. The Dll4- Hey2- EphrinB2 signaling cascade facilitates arterial differentiation where as venous differentiation is promoted by COUP-TFII signaling [6, 7].

The later stage of vascular development includes blood vessel maturation. A reciprocal interaction established between endothelial cells and smooth muscle cells is essential for vessel maturation. This interaction, recruits smooth muscle cells around the vascular tube and thus stabilize the blood vessel. *TGF-β* and *PDGF- β* are two major genes involved in this process. TGF-β has two types of receptors, type I activin-like kinase (ALK) and type II serine/threonine kinase receptors. Both type1 and type II receptors of TGF-β interacts with type III co-receptor, Endoglin. Type I receptor, ALK and the co-receptor Endoglin are essential for promoting smooth muscle cell recruitment. Arterio-venous identity is lost in both ALK and Endoglin null embryos. Another paracrine signaling established between smooth muscle cells and endothelial cells through platelet- derived growth factor B (PDGF- β) also advances smooth muscle cell recruitment [8, 9]. Recently it has been reported that KLF2, a transcription factor is involved in inducing the recruitment of smooth muscle cells by PDGF- β [10].

In the normal brain, nervous and vascular systems maintain an inseparable relationship at cellular, molecular and anatomic levels [11–13]. Endothelial cells of brain capillaries are encircled by astrocytic end feet [14–16] and astrocytic interaction induces brain capillary endothelial cells to express certain proteins such as GGTP and GLUT1 [16–18]. An interesting feature of AVM is the absence of a normal capillary system between arteries and veins and replacement by a malformed vascular structure (nidus).

In this report, we present the result of a comprehensive analysis of expression of genes involved in both early (*HEY2*, *DLL4*, *EFNB2*, *COUP-TFII*) and late phase of vascular development (*ENG* and *KLF2*), in arteriovenous malformations in the brain. We also investigated whether AVM nidus expresses brain capillary specific proteins such as GGTP and GLUT1.

## Materials and methods

### Study subjects and ethical approval

Brain AVM tissue samples were collected from 10 patients who underwent excision of the lesion between March 2014 and December 2015 at Department of Neurosurgery, Sree Chitra Tirunal Institute for Medical Sciences & Technology (SCTIMST). Ethical approval was obtained from Institutional Human Ethics Committees of SCTIMST (SCT/IEC/629/JUNE-2014) and Rajiv Gandhi Centre for Biotechnology (IHEC/01/2015/05), Thiruvananthapuram. Written informed consents were obtained from the study patients prior to surgery for the collection of tissue samples.

Patients were in the age group of 22–35 years. Clinical profile of patients is given in Table 1. Patients with Hereditary Hemorrhagic Telangiectasia (HHT) were excluded from the study. Cerebral AVM was confirmed by cerebral angiography and MRI scan. Brain samples from 10 patients, of age range 25–31 years who were operated at SCTIMST for temporal lobe epilepsy served as controls.

**Table 1:** Clinical data of patients with brain arteriovenous malformation.

SL No	Age	Sex	Location of AVM lesion	S-M Grade	Presenting symptoms
1	25	M	Parietal	2	Seizures
2	33	F	Temporal	2	Head ache
3	22	F	Parietal	2	Seizures
4	25	M	Frontal	2	Seizures
5	24	M	Parietal	2	Seizures
6	35	M	Frontal	1	Head ache and vomiting
7	24	M	Frontal	2	Head ache
8	32	M	Frontal	2	Seizures
9	26	M	Frontal	2	Seizures
10	28	M	Temporal	2	Memory loss and behavioral problems

M: Male, F: Female, S-M Grade: Spetzler-Martin Grade

### Tissue specimen

AVM and control brain tissues were divided into three parts and stored in 10% neutral buffered formalin, 4% Paraformaldehyde (PFA) and RNA later solution. Tissues stored in 10% neutral buffered formalin were used for histological, immunohistochemical analysis and those stored in 4% PFA were used for immunofluorescence analysis. Proteins were isolated from tissues stored in RNA later solution.

### Hematoxylin-Eosin (H & E) staining, elastin Van-Gieson (EVG) staining and Immunohistochemistry (IHC)

After two days of formalin fixation, tissues were processed and embedded in paraffin. Tissue sections of 5 µm thickness were cut from paraffin blocks and used for H & E staining [19], EVG staining [20] and immunohistochemistry. IHC supersensitive kit from Biogenex was used for immunohistochemical analysis. Peroxide blocking was done in hydrogen peroxide to quench endogenous peroxidase activity. Antigen retrieval was done by incubating the sections in citrate buffer at 95°C for 20 min. Primary antibodies used were anti α-SMA antibody (Mouse, 1:10000; Abcam, UK), anti PECAM-1 antibody (Mouse, 1:100; Abcam, UK), anti Hey2 antibody (Rabbit, 1:300; Sigma-Aldrich, India), anti Dll4 antibody (Rabbit, 1:1000; Abcam, UK), anti EphrinB2 antibody (Rabbit, 1:200; Santa Cruz, USA), anti COUP-TFII antibody (Rabbit, 1:600; Abcam, UK), anti Endoglin antibody (Rabbit, 1:500; Santa Cruz, USA), anti GGTP antibody (Rabbit, 1:700; Abcam, UK) and anti GLUT1 antibody (Rabbit, 1:1000; Abcam, UK). Incubation with primary antibody was done for overnight at 4°C. Excess antibody was washed in tris—buffered saline (TBS) and followed by addition of secondary antibody conjugated horse radish peroxidase. Later 3, 3’-diaminobenzidine was added and sections were incubated for 2–5 min. Counter staining was done with hematoxylin. Excess stain was washed off, sections dehydrated in ascending grades of IPA, cleared in xylene and mounted in dibutyl-phthalate xylene. A secondary antibody only staining was done to specify the IHC results. Microscopic examination was done using a light microscope (Nikon Eclipse 55i microscopic system, Japan). Images were collected using 10X, 20X and 40X magnification objectives.

### H-score analysis

We have done IHC scoring by manual method, as it was reported to be as significant as automated methods of IHC analysis [21]. H-scoring was done as described elsewhere [22]. Five fields were randomly selected for every test and control samples. Scoring was done on a scale ranging between 0 (no staining) - 3 (maximum staining). H scoring was done using formula (1+i) pi where “i” is the intensity score and “pi” is the percentage of the cells with that intensity. IHC scoring was done in all the cells of blood vessels in AVM and control tissues. Brain parenchyma cells were excluded from the IHC analysis.

### Immunofluorescence analysis

After overnight fixation of tissues in 4% PFA, tissues were suspended in increasing concentrations of sucrose in phosphate buffer saline (10%, 20%, and 30%). Tissues were embedded in Optimal Cutting Temperature Compound (OCT) and were stored at -80°C. Later, sections of 5 µm thickness were taken using a cryotome. On the day of the staining, microscopic slides with tissue sections were fixed in acetone for one minute. Antigen was retrieved in citrate buffer, non specific binding was prevented by blocking with 3% bovine serum albumin (BSA). Immunofluorescence staining was attempted for KLF2 (Rabbit, 1:200; Abcam, U.K) antibody. Primary antibody incubation was followed by addition of Alexafluor conjugated rabbit secondary antibody (1:500, Abcam, U.K). Nuclear staining was done using Hoechst 33342 (1:500, Sigma-Aldrich, India). Later, sections were mounted in fluorochrome and excited for fluorescence using a fluorescent microscope (Leica SP2 laser scanning spectral confocal system, 60X magnifications).

### Western blot analysis

Proteins were isolated from whole tissue using radio immunoprecipitation assay (RIPA) buffer as described earlier [22]. Protein concentration was determined with Bradford reagent (Bio-Rad, USA). 30 μg/µl of protein was loaded on 12% SDS-PAGE gel and electrophoresis was done. Later, proteins were transferred to a nitro cellulose membrane. Nonspecific binding sites were blocked by placing the membranes in 5% BSA in TBS for 1 h. Primary antibody was added and incubated at 4°C for overnight with mild shaking. Primary antibodies in this study were used in following dilutions, anti Hey2 antibody (1:500), anti Dll4 antibody (1:1000), anti Ephrin B2 antibody (1:500), anti COUP-TFII antibody (1:800), anti Endoglin antibody (1:1000), anti KLF2 antibody (1:1000), anti GGTP antibody (1:1000) and anti GLUT1 antibody (1:1000). Subsequently, membranes were probed with horse radish peroxidase (HRP) conjugated secondary antibodies (Anti-rabbit (1:5000), Abcam, U.K and Anti-mouse antibody (1:5000), Abcam, U.K). Finally, the bands were detected using enhanced chemiluminescence (ECL) (Bio-Rad) and the band was transferred to Kodak X-ray film.

### Statistical analysis

All results are expressed as mean ± SEM. Graph Pad PRISM version 6.07 was used for statistical analysis. Student’s t- test was done to analyze difference in protein expression levels. P value less than 0.05 was considered as statistically significant.

## Results

### H &E, EVG, α-SMA and PECAM-1 staining

**Fig 1:**
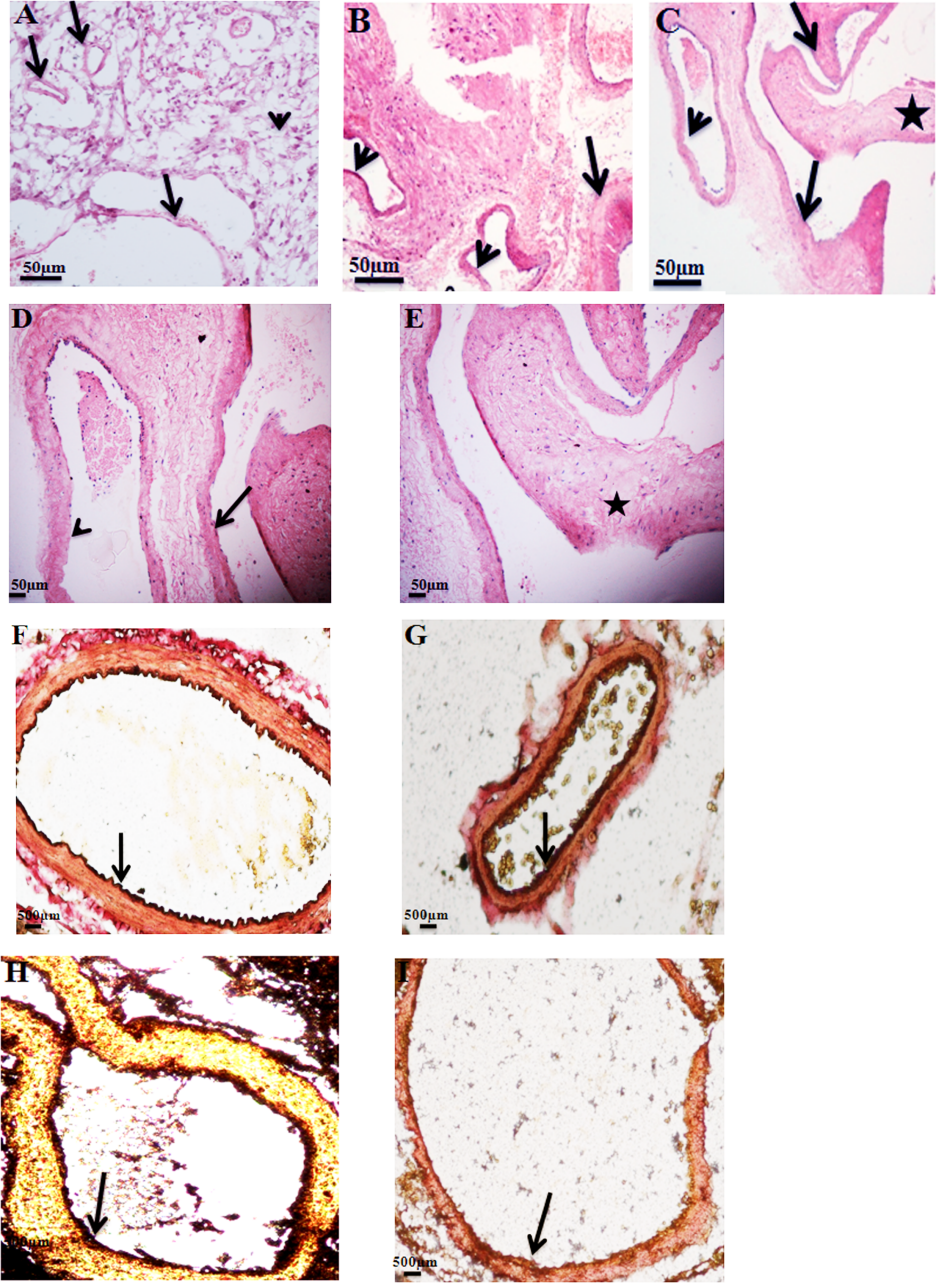
(A) Hematoxylin and Eosin staining of control tissues and (B, C, D and E) AVM nidus. (A) H & E staining of control brain reveals normal histological pattern of brain parenchyma (arrowhead) and blood vessels (arrow). (B) H & E staining of AVM nidus reveals that nidus consists of blood vessels of different size and morphology. There are both thin walled (arrowheads) and thick walled vessels (arrow). (C) Partial denudation of endothelial cell layer is seen (arrowhead). Large sized veins (arrows) with uneven thickness of tunica media layer are seen. Thickened tunica media layer (asterisk) is seen in AVM vessels. Magnification 10X. (D) Higher magnification image of AVM nidus clearly demonstrates partial denudation of endothelial cell layer (arrowhead) and large sized vein (arrows) with an uneven thickness of tunica media layer. (E) A blood vessel with thickened smooth muscle cell layer (asterisk) is seen. Magnification 20X. **(F and G) EVG staining of control vessels and (H and I) AVM nidus structures.** (F) A control artery with a thick internal elastic lamina layer (arrow) is depicted. (G) Staining for elastic fibres (arrow) in the wall of a normal vein is evident. (H) Dark brown colour (arrow) indicates the internal elastic lamina layer of a large artery in AVM nidus. (I) A dysplastic vein in AVM nidus with a thin internal elastic layer (arrow) is shown. Magnification 10X.

In control tissues, blood vessels and the surrounding neural tissue had normal histological features (Fig 1). Histological analysis of AVM nidus revealed that nidus consists of heterogeneous vascular structures with distinct morphological features. There were medium and small sized vessels. However we observed that majority of vessels in nidus were large sized vein with an uneven thickness of tunica media layer. Partial fragmentation of endothelial cell layer was also observed. In a subset of AVM vessels thickening of smooth muscle cell layer was seen (Fig 1).

In the control brain tissue, we observed arteries and veins with normal distribution of elastic lamina layer **(**Fig 1). In AVM nidus, EVG staining helped in delineating arteries and veins (Fig 1). Internal elastic lamina layer was the distinct characteristic of arteries from veins. Large arteries with harder media structure and thick internal elastic lamina layer were observed in AVM nidus. We observed dysplastic veins in AVM nidus with less staining for elastic fibers.

**Fig 2:**
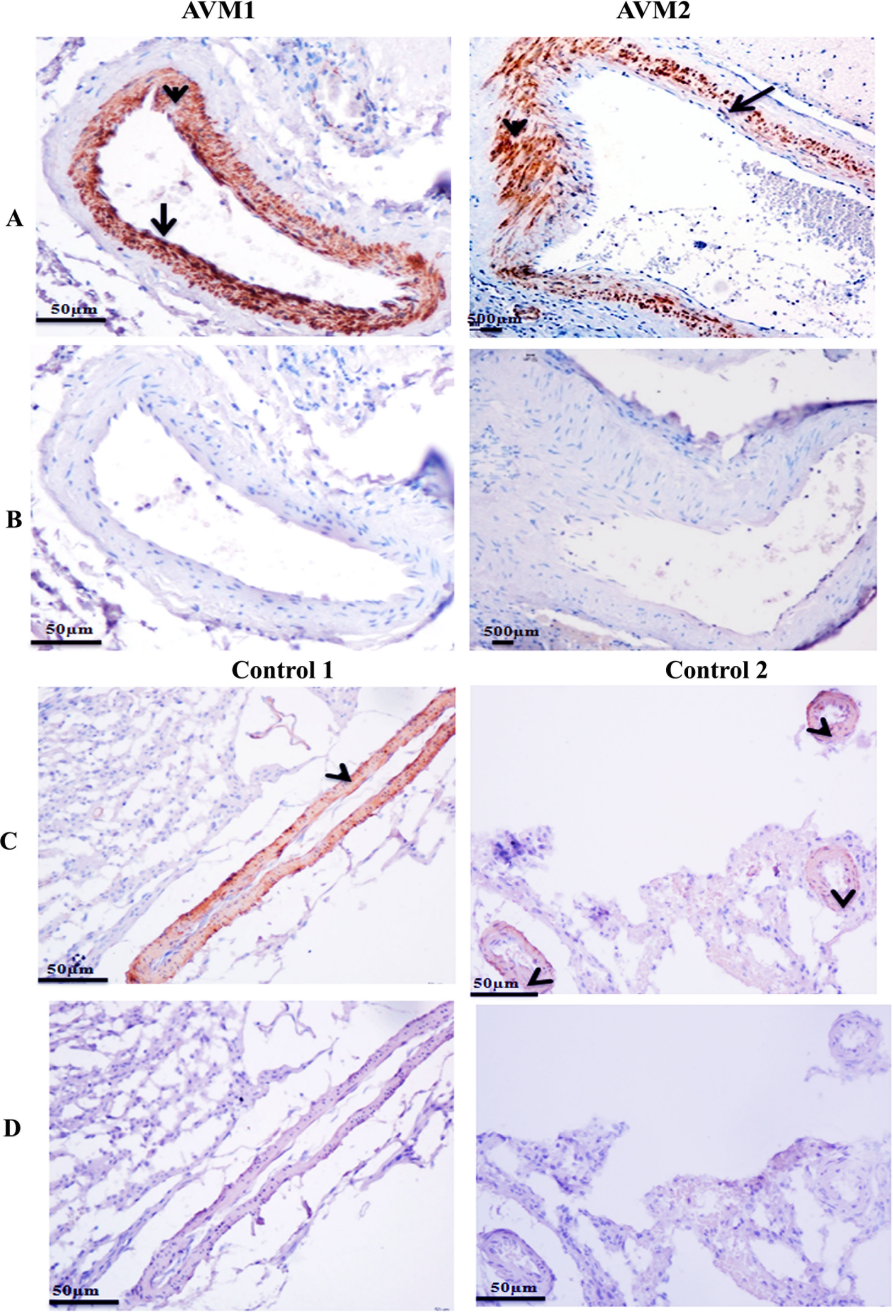
Immunohistochemical staining of α-SMA in (A) AVM nidus structures and (C) control tissues. (A) There is intense staining of α-SMA in the endothelial cell lining (arrow) and in the peri-endothelial cell layer (arrowhead) of AVM vessels (AVM 1). AVM nidus structures consist of both circular (arrow) and longitudinal bundles (arrowhead) of smooth muscle cells (AVM 2). (C) α-SMA is expressed in the smooth muscle cells of control vein (control 1) and control arteries (control 2). (B and D) Secondary isotype controls for AVM and control tissues respectively. Magnification 20X.

There was heterogeneous pattern of expression of α-SMA in AVM nidus structures (Fig 2). α-SMA staining was seen in endothelial cell layer and sub endothelial layers of AVM nidus (AVM1). α-SMA staining revealed the presence of both circular and longitudinal bundles of smooth muscle cells in AVM nidus (AVM2). We identified less expression of α-SMA in the tunica media layer of a few AVM vessels (S1A Fig). Higher magnification images demonstrating circular and longitudinal smooth muscle cells are shown in S1 Fig. α-SMA staining was observed in the smooth muscle cell layer of control veins and arteries (Fig 2). Secondary isotype controls confirmed the absence of non-specific staining (Fig 2).

**Fig 3:**
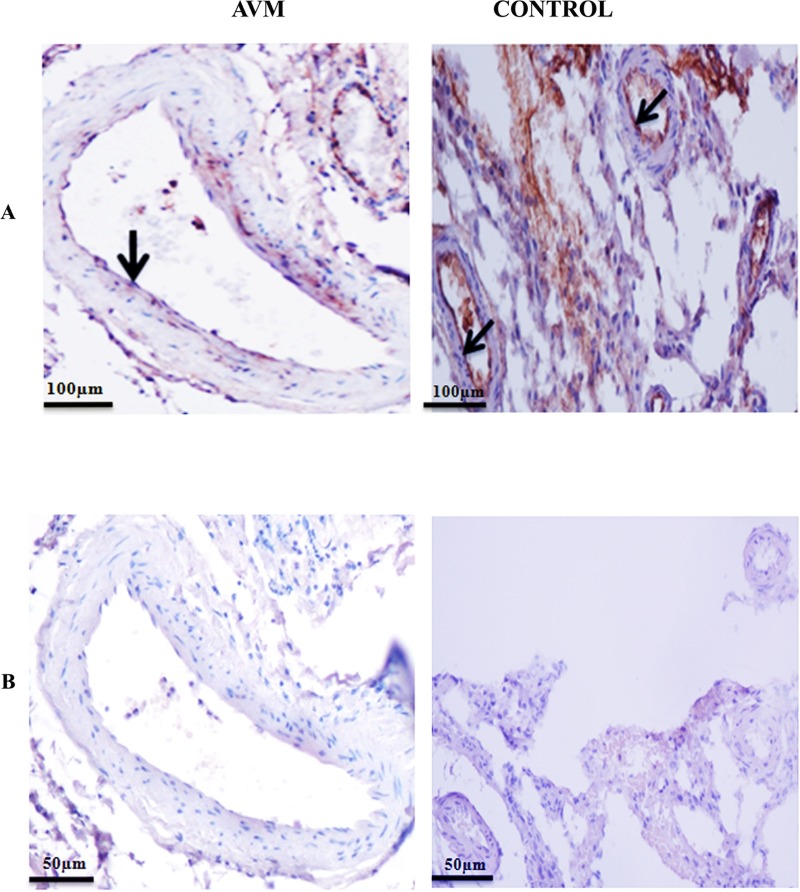
Immunohistochemical staining of PECAM-1 antibody in AVM nidus structures and control tissues. (A) PECAM-1 is expressed in the endothelial cell lining of (arrow) AVM and control vessels. (B) Photomicrograph of tissues stained with secondary antibody alone Magnification 20X.

PECAM-1 (platelet endothelial cell adhesion molecule-1) also known as CD-31 is an endothelial cell specific marker. In our study, we observed that it is specifically expressed in the endothelial cell lining of AVM and control vessels (Figs 3 and S1E). Secondary isotype controls are shown in Fig 3.

Immunohistochemical staining with α-SMA and PECAM-1 antibodies in AVM vessels revealed that endothelial cells of certain AVM vessels express α-SMA (Fig 2, AVM1 and Fig 3, AVM respectively).

### Analysis of arterial differentiation markers (Hey2, Dll4, EphrinB2) in AVM and control vessels

Immunostaining was carried out to analyze the expression pattern of Hey2, Dll4 and EphrinB2 in AVM and control vessels. Hey2 expression was restricted to the nucleus where as EphrinB2 and Dll4 were localized to the cytoplasm. Nidus from brain AVM of all the ten patients had the expression of all the three arterial markers Hey2, Dll4, EphrinB2. Pattern of staining of these markers in the large and small blood vessels of AVM nidus were heterogeneous.

**Fig 4:**
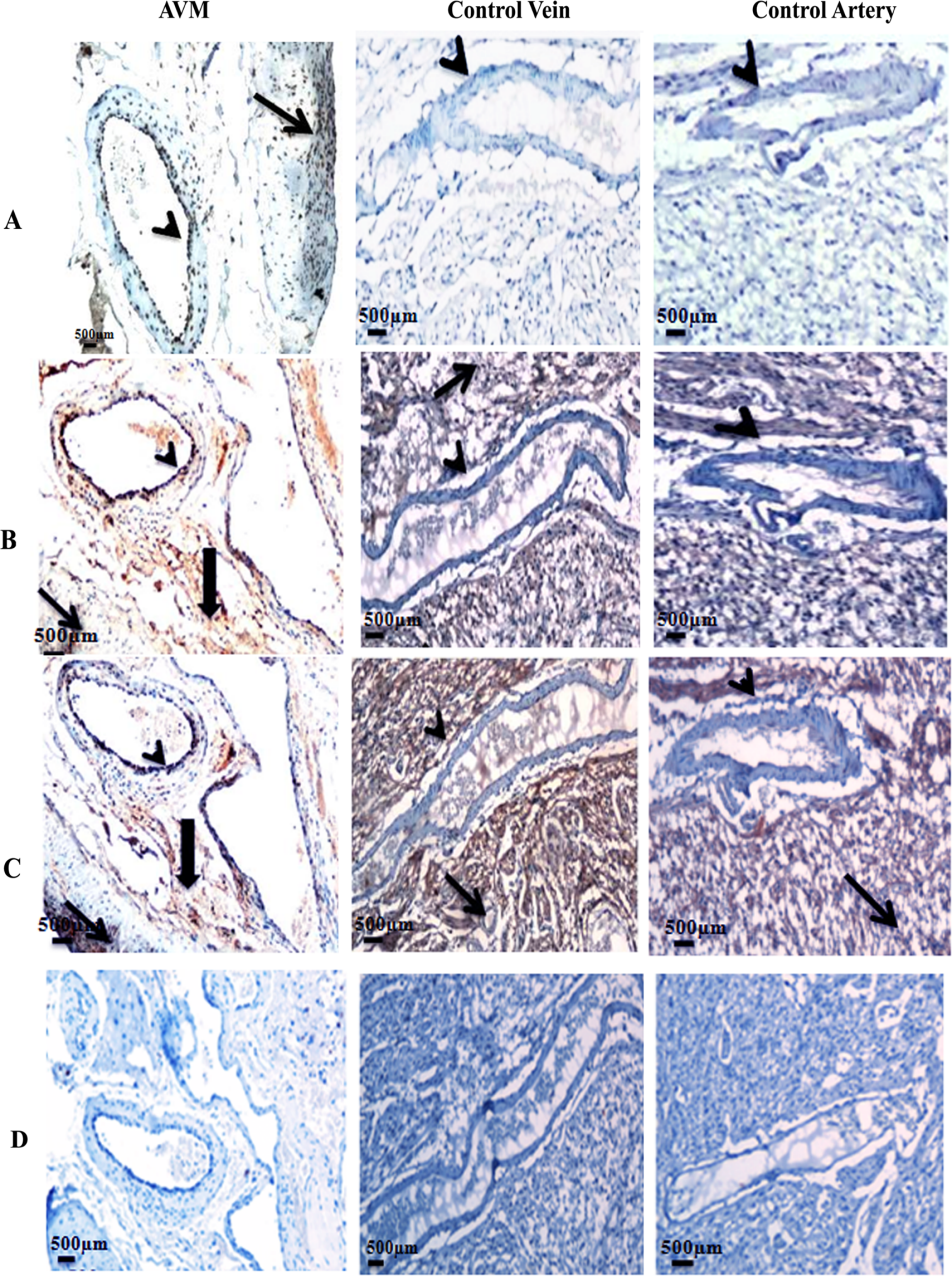
Representative photomicrographs of immunohistochemical analysis of arterial markers (A) Hey2, (B) Dll4 and (C) EphrinB2 in AVM and control tissues obtained from temporal lobe epilepsy patients. (A) There is intense staining of Hey2 in AVM nidus structures compared to control vessels. Hey2 is expressed in the endothelial cell lining (arrowhead) and smooth muscle cells (thin arrow) of AVM nidus structures. In control vessels (arrowhead), the Hey2 expression is much less. (B) In AVM nidus, Dll4 is expressed with high staining intensity in the endothelial cell lining (arrowhead), smooth muscle cells (thin arrow) and tunica adventitia layer (thick arrow). There is much less expression of Dll4 in blood vessels in control brain (arrowhead). In control tissue, Dll4 positive cells are found in adjacent brain parenchyma cells (thin arrow). (C) Nidus of AVM has high expression of EphrinB2 in tunica intima (arrowhead), tunica media (thin arrow) and tunica adventitia (thick arrow) layer of blood vessels. In control tissue, brain parenchyma expresses EphrinB2 positive cells (arrow) and blood vessels have much less expression of EphrinB2 (arrowhead). (D) Photomicrograph of tissue stained with secondary antibody alone. 10X magnification.

Hey2 was highly expressed in the endothelial cell lining of AVM vessels compared to control vessels. The smooth muscle cells in tunica media layers of the AVM vessels had an augmented Hey2 expression when compared to control vessels. In control brain tissues, both arteries and veins had very low Hey2 expression (Fig 4). A higher magnification image representing HEY2 expression in AVM vessel is given in S2A Fig. Hey2 expression was present in arterioles of control brain tissues. We observed expression of Hey2 in surrounding brain parenchyma of 80% control tissues (S2B Fig). Dll4 was localized in the cytoplasm and highly expressed in the cells of tunica intima, media and adventitial layers of AVM nidus compared to control vessels (Fig 4). Expression of Dll4 was observed in the brain parenchyma of 80% of control tissues. EphrinB2 was highly expressed and localized in the cytoplasm of cells of all the three tunics of AVM vessels compared to control vessels (Fig 4). Differences in expression were statistically significant for all the three arterial markers analysed (P < 0.0001). Tissue sections were stained with secondary antibody alone that confirmed the absence of nonspecific staining in our IHC studies (Fig 4D).

### Analysis for vein differentiation marker COUP-TFII

**Fig 5:**
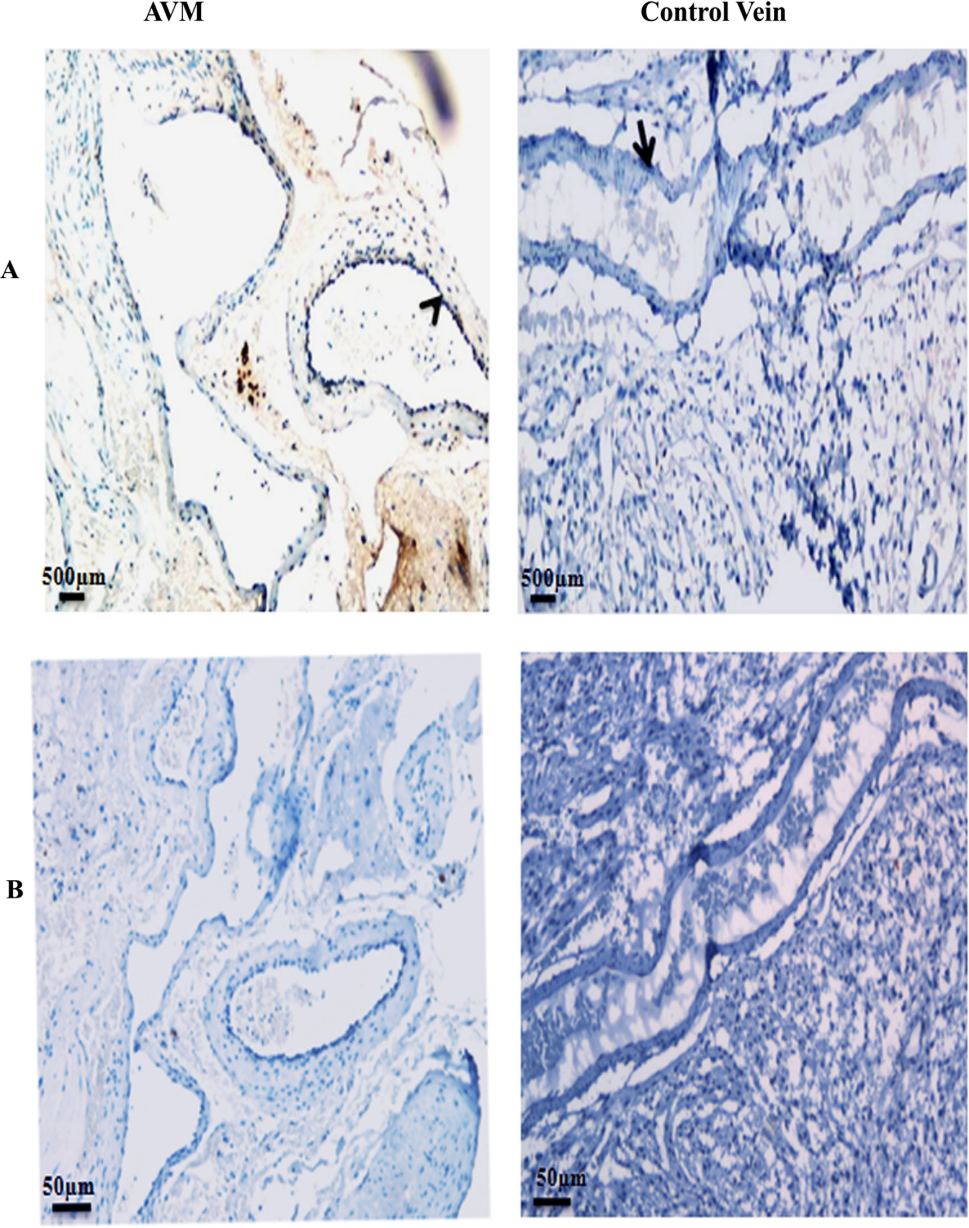
Photomicrograph of tissues stained with the antibody against COUP-TFII in AVM and control. (A) There is intense staining of COUP-TFII in AVM nidus structures compared to control tissues. COUP-TFII (arrowhead) is localized in the endothelial cell lining of AVM vessels. COUP-TFII (arrow) is not expressed in the blood vessel in control tissue. (B) Photomicrograph of tissue stained with secondary antibody alone. 10X magnification.

Expression pattern for COUP-TFII in AVM nidus structures and veins in control tissues was examined by immunohistochemistry (Fig 5). COUP-TFII was localized to the nucleus and predominantly seen in the endothelial cell lining of AVM nidus structures. This pattern of COUP-TFII distribution was observed in nidus of AVM tissues of 8 patients. A higher magnification image for COUP-TFII expression in AVM nidus is given in S2C Fig. There was significant increase (P < 0.0001) in the expression of COUP-TFII in AVM compared to control veins. COUP-TFII expression was very low in the blood vessels as well as brain parenchyma of all the ten control tissues. Secondary isotype controls are shown in Fig 5.

### Expression of GLUT1 and GGTP in AVM and control vessels

**Fig 6:**
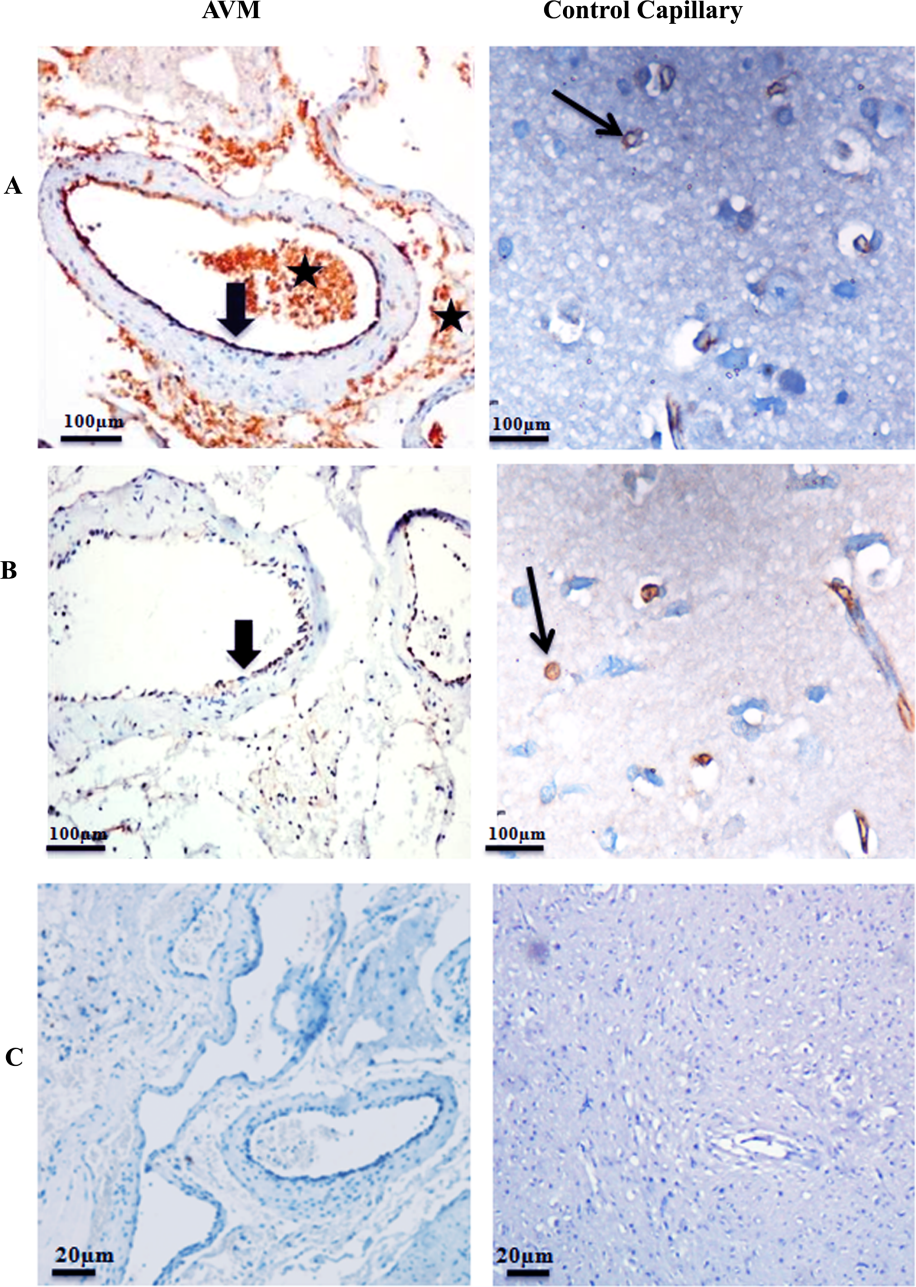
Photomicrograph of AVM and control tissues stained with antibodies against (A) GLUT1 and (B) GGTP. (A) GLUT1 is highly expressed in the endothelial cell lining of AVM nidus (thick arrow). GLUT1 is expressed in red blood cells adjacent to the AVM tissue (asterisk). GLUT1 is expressed with high intensity in capillaries of control tissue (thin arrow). (B) There is intense staining of GGTP in the endothelial cell lining of AVM vessels (thick arrow). GGTP is expressed with high intensity in the control capillaries (thin arrow). 20X magnification. (C) Secondary isotype controls.

GLUT1 and GGTP are transmembrane proteins and were expressed in the endothelial cell lining of AVM vessels. GLUT1 and GGTP were highly expressed in both small and large blood vessels of AVM nidus structures of all ten patients, compared to control vessels. In control brain tissues, we observed expression of GLUT1 and GGTP in capillaries (Fig 6). Large vessels such as arteries in control brain had less expression of these proteins as expected (S2 Fig). There was no expression of brain capillary marker GGTP and much less expression of the other capillary marker GLUT1 in large vessels in control brain. A difference in expression between AVM nidus and large vessels in control brain were highly significant for GGTP and GLUT1 (P < 0.0001). Secondary isotype controls confirmed the absence of non-specific staining (Fig 6).

### Expression of endoglin and KLF2 in tissue specimens

**Fig 7:**
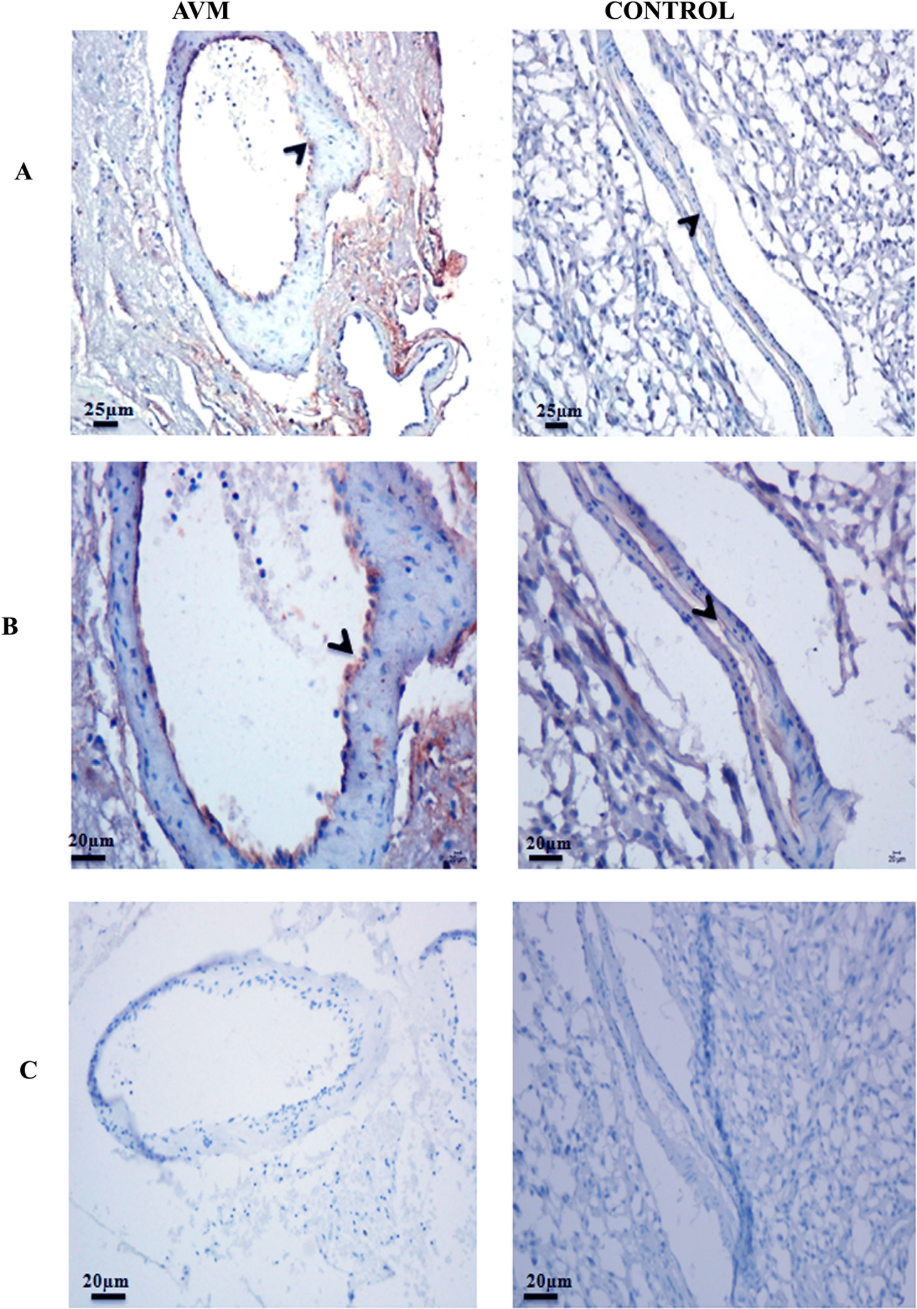
Photomicrograph of tissues stained with the antibody against endoglin in AVM and control. Endoglin expression is localized in the endothelial cell lining (arrowhead) of AVM and control vessels. Endoglin is expressed with high staining intensity in AVM vessels whereas control vessels have a weak expression of this protein. (A) panel shows 20X magnification and (B) panel shows 40X magnification. (C) Secondary antibody controls (20X magnification).

The expression pattern of endoglin was assessed in AVM and control tissues using immunohistochemistry. Fig 7 represents Endoglin expression in AVM and control (20X magnification) and Fig 7 represents the staining pattern in 40X magnification. In both AVM and control vessels, Endoglin was localized in endothelial cells. Endoglin was significantly over expressed in AVM nidus compared to control vessels (P < 0.0001). We observed a distinct pattern of expression of Endoglin in control vessels. Endoglin was intensely expressed in micro vessels of control tissues rather than in large vessels (S2F Fig). Secondary antibody staining controls are given in Fig 7.

**Fig 8:**
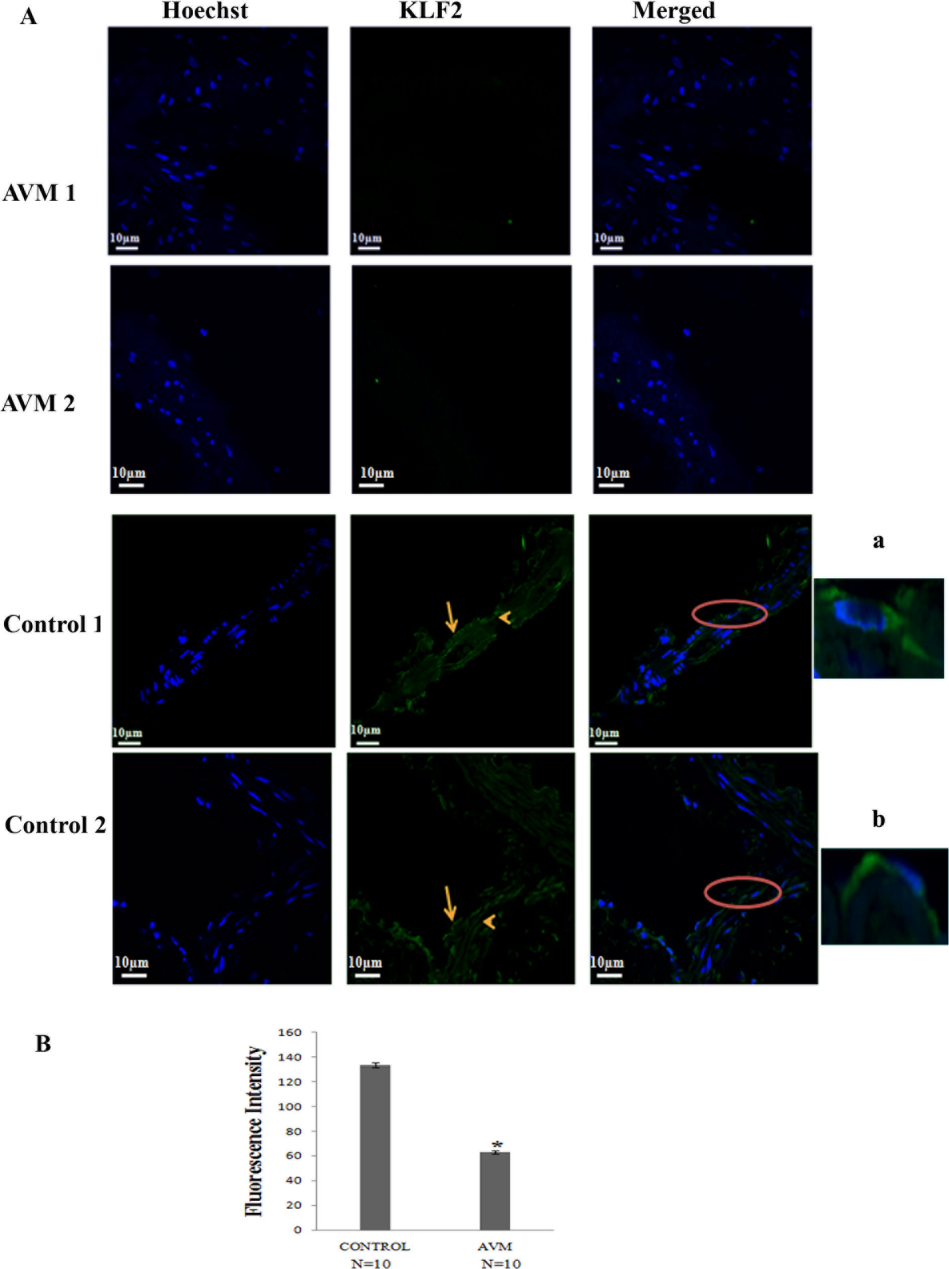
**(A) Photomicrograph of tissues stained with the antibody against KLF2 in AVM and control tissues.** KLF2 is not expressed in AVM nidus structures. In control vessels, KLF2 is expressed (green) in the endothelial cell lining (arrowhead) and smooth muscle cells (arrow). Magnified images are shown in a and b. Hoechst 33342 (blue) is used to counter stain nuclei. 60X magnification. **(B) Graphical representation of fluorescence intensity of KLF2 in AVM and control tissues.** KLF2 expression is significantly low in AVM nidus structures (n = 10) compared to control tissues (n = 10) (*P < 0.001).

Immunofluorescence staining was done to analyze KLF2 expression in AVM and control vessels (Figs 8 and S3). Immunofluorescence intensity was quantified using microscope imaging software, NIS-Elements Viewer. Expression of KLF2 in AVM nidus was significantly less compared to control (P <0.001) (Fig 8B). 70% of vessels in nidus structures lacked KLF2 expression and in 30% of vessels, expression was low. This pattern of KLF2 expression was observed in all AVM samples analysed. In this study, KLF2 was expressed in endothelial cell lining as well as in smooth muscle cells in control vessels.

**Fig 9:**
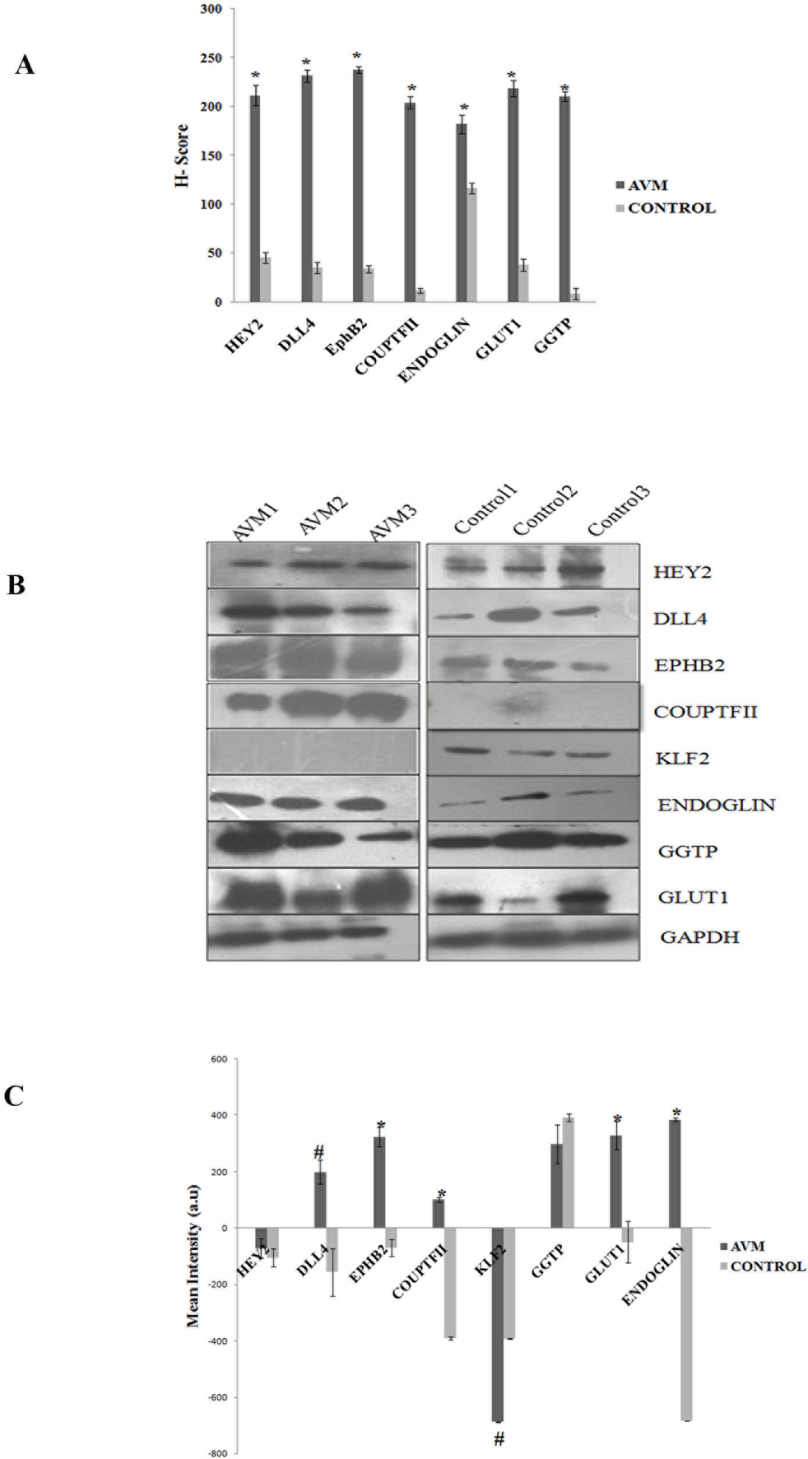
**(A) H-score analysis of vascular differentiation (Hey2, Dll4, EphrinB2 and COUP-TFII), vascular maturation (Endoglin) and brain capillary markers (GLUT1 and GGTP) in AVM and control tissues.** Expression of Hey2, Dll4, EphrinB2, COUP-TFII, Endoglin, GLUT1 and GGTP are significantly increased in AVM nidus structures (n = 10) compared to blood vessels in control tissues (n = 10). Statistically significant (*P < 0.0001) when compared to control vessels. H-Score was calculated by formula (1+i) pi; “i” is the intensity score and “pi” is the percentage of the cells with that intensity. **(B) Western blot analysis of vascular differentiation, vascular maturation and brain capillary marker proteins. (C) Densitometric analysis of western blot band of Hey2, Dll4, EphrinB2, COUP-TFII, GLUT1 and GGTP in AVM and control tissues.** Dll4, EphrinB2, COUP-TFII, Endoglin and GLUT1 expression are significantly increased in AVM tissues compared to control tissues. The KLF2 expression is significantly decreased in AVM compared to control. Statistically significant (*P < 0.0001 and ^#^ P < 0.001). There is no statistically significant difference in expression of Hey2 and GGTP in AVM tissues compared to control tissues. GAPDH is the loading control. AVM (n = 5) and control (n = 5).

Proteins such as Hey2, Dll4, EphrinB2, GGTP, GLUT1, COUP-TFII and Endoglin were analysed by immunohistochemistry, quantified manually by H-score analysis and illustrated in Fig 9. H-score analysis revealed a statistically significant upregulation of all the proteins analysed (*P < 0.0001).

### Western blot analysis

Results of western blot analysis were consistent with the findings from immunohistochemistry. We were able to detect bands corresponding to all the proteins analysed both in AVM and control tissues, except for COUP-TFII in control and KLF2 in AVM nidus (Fig 9). Densitometry analysis (Fig 9C) indicated significant up-regulation of Dll4, EphrinB2, COUP-TFII, GLUT1and Endoglin in AVM structures compared to control (P < 0.0001) and P < 0.001). There was no significant intensity difference with regards to densitometric analysis of Hey2 and GGTP bands in AVM compared to control tissues. Considering the results of IHC, this contrasting result in immunoblots can be due to the presence of Hey2 and GGTP in control brain parenchyma as well. KLF2 was significantly down-regulated in AVM structures compared to control (P < 0.001).

## Discussion

Our study was aimed to characterize the nature of vascular structures of cerebral arteriovenous malformations (AVM). We found differential expression of genes involved in the early and late stage of vascular development in these malformed vessels.

Vascular development consists of an early stage of vasculogenesis and a later stage of vascular maturation. The fate of an endothelial cell, either to be an artery or to be a vein is determined in the early stage of vascular development [4, 5]. Expression of specific markers in endothelial cells at this stage determines the fate of the endothelial cell. Arterial fate is determined by Dll4 -Hey2- EphrinB2 pathway and vein fate by COUP-TFII [6, 7]. Later stages of vascular development involve the formation of the mature blood vessel. Blood vessel maturation is facilitated by two genes *ENG* and *KLF2* [8–10].

Histological analysis showed that AVM nidus is a collection of aberrant vascular structures depicted as hypertrophic veins: with increased thickness of media layer; arterial like structures with reduced tunica media layer and small arteriole and venules like vessels. In AVM nidus, EVG staining revealed the presence of large arteries and dysplastic veins with discontinuous internal elastic lamina layer.

α-SMA staining demonstrated aberrancy in the smooth muscle layer of AVM associated blood vessels. We observed that endothelial cells of certain AVM vessels expressed α-SMA. We confirmed our above finding by staining those blood vessels with PECAM-1, a membrane glycoprotein specifically expressed in endothelial cells. Moreover, there are previous reports on α-SMA expression in endothelial cells [23]. In certain AVM vessels there was much less expression of α-SMA in the tunica media layer. Previous studies have reported that when vascular smooth muscle cells are in the proliferative stage there is a loss of expression of α-SMA [24].

### Both artery and vein differentiation markers are expressed in AVM nidus structures

EphrinB2 is the key determinant of arterial endothelial cell fate [6]. The EphrinB2 expression is induced by Dll4-Notch-Hey2 signaling [25]. This pathway is tightly regulated in vascular development and any change in its regulation can lead to vascular abnormalities [26–31]. In our study, we observed an augmented expression of EphrinB2, Hey2 and Dll4 in the nidus structures from patients with cerebral AVM when compared to normal brain arteries. Previous studies have reported that Notch-1 and Notch-3 are over expressed in AVM nidus [32–34]. Together our findings suggest that a deregulated arterial specification signaling might have a significant role in the pathogenesis of AVM. We observed much less expression of EphrinB2, Dll4 and Hey2 in normal mature cerebral arteries. EphrinB2, Hey2 and Dll4 have roles only in the embryonic stages of vascular development [32].

Vascular fate of an endothelial cell for venous differentiation is determined by COUP-TFII [35, 36, 37]. We observed an increased expression of COUP-TFII in AVM nidus structures compared to control cerebral veins. Expression of vein related genes in nidus structures of AVM have been recently reported [38].

It is well known that Notch signaling and subsequent arterialisation pathways are suppressed by COUP-TFII [36, 37]. In HUVEC cells, COUP-TFII is known to bind to the Hey2 promoter and inhibit its expression. Knockdown of the *COUP-TFII* gene leads to over expression of Notch signaling pathway genes [37].

Previous studies had identified that endothelial cell of AVM vessels expresses either artery or vein marker [32, 33, 34]. Co-expression of both artery-specific genes *HEY2*, *DLL4*, *EFNB2* and vein specific gene *COUP-TFII* in endothelial cells of AVM vessels as observed in our study is a novel finding. Simultaneous expression of these genes in the endothelial cells of the nidus could be the basis for deregulation of artery-vein fate determination in AVM. We speculate that in the endothelial cells of AVM, default mechanisms of artery and vein fate determination are disrupted and result in the genesis of blood vessels, which are neither arteries nor veins. A recent report on defective characteristics of AVM derived endothelial cells supports our hypothesis [39, 40].

### AVM structures express brain capillary markers

A critical feature of AVM is that it is characterized by a complex nidus structure, instead of a normal capillary system [1]. To understand whether a normal capillary system is replaced by nidus structures in AVM, we analysed the expression of specific capillary markers. There are no specific markers to identify capillaries [41, 42]. An interesting feature of brain capillaries is that they express certain proteins such as GGTP and GLUT1 unlike capillaries in other anatomical locations [17, 18, 43]. In control tissues, we confirmed that GGTP and GLUT1 are specifically expressed in capillaries rather than in large vessels. Interestingly, AVM nidus structures also express brain capillary proteins. There is a recent study by Murphy PA *et al* where they reported that AVM nidus structures especially AV shunts can be formed from preexisting normal capillaries. They identified that Notch4 over expression in neonatal mice leads to the formation of AV shunts from normal capillaries [44]. On another note, previous studies have reported giant bed capillaries in brain tissue surrounding the nidus [45–48]. Expression of GGTP and GLUT1 in brain capillaries is induced by Astrocytes [17, 18, 43]. Persistence of brain capillary proteins in AVM nidus structures can hence be explained due to astrocytic interactions. Taken together, the expression of brain capillary proteins in AVM nidus suggests an initial capillary phenotype of nidus structures. We speculate the involvement of multiple secondary insults in the conversion of a normal capillary system to AVM nidus.

### Vascular maturation genes are differentially expressed in AVM

Endoglin maintains vascular integrity by promoting recruitment of vascular smooth muscle cells towards the endothelial cell network [8, 9]. In a hereditary form of AVM, associated with a mutation in the *ENG* gene, there is a 50% reduction of Endoglin in AVM blood vessels compared to normal vessels [49]. Interestingly in our study on sporadic brain AVM, we observed an increased expression of Endoglin in AVM nidus structures compared to control vessels. A previous study had reported that over expression of Endoglin in endothelial cells can disrupt endothelial-smooth muscle cell interactions and manifest malformed vessels [50]. An earlier study on sporadic AVM had reported expression of Endoglin in AVM vessels [49]. However, they did not observe an increase in expression of Endoglin in AVM compared to control blood vessels as we identified.

KLF2 induces recruitment of smooth muscle cells by PDGF- βduring vascular maturation. KLF2 deficient embryos have normal endothelial cell development pattern. Smooth muscle cell recruitment is defective in these embryos and thus vascular integrity is affected [10]. A novel yet intriguing observation in our study is the highly reduced expression of KLF2 in nidus structures compared to normal vessels. KLF2 is not involved in forming primitive endothelial cell tube and does not influence any of the genes involved in early stages of vascular development [10]. Thus KLF2 can be considered as a marker particular to blood vessel maturation and absence of KLF2 in AVM confirms the immature nature of the AVM structures. Expression of *KLF2* gene is reduced in conditions of disturbed flow of blood when compared to conditions of normal laminar flow [51, 52]. As the laminar flow of blood is not maintained in AVM nidus, we speculate reduction of KLF2 in AVM lesions is contributed by disturbed blood flow.

## Conclusion

Our study demonstrates that AVM structures have overlapping features of vascular identity. AVM nidus structures express markers specific to arteries and veins as well as capillaries. Genes involved in vessel maturation are also differentially expressed in AVM structures compared to control vessels. Thus AVM vessels can be considered as aberrant vessels, which are not terminally differentiated and inadequately matured possibly because of a developmental arrest. This study, to our knowledge, is the first comprehensive gene expression analysis of vascular structures of AVM.

## Supporting information

S1 Fig Immunohistochemical staining of (A, B, C, D) α-SMA and (E and F) PECAM-1 antibody in AVM nidus. (A) α-SMA staining is seen in the endothelial cell lining of AVM nidus (arrow) and there is much less expression of α-SMA (arrowhead) in tunica media layer. 20X magnification. (B, C, D) 40X magnification images of α-SMA staining showing circular (arrow) and longitudinal smooth muscle cells (arrowhead) in AVM vessels. (E) PECAM-1 is expressed in the endothelial cell lining of AVM vessels. (B) Secondary isotype controls for PECAM-1 staining. 20X magnification.
(TIF)

S2 Fig Immunohistochemical staining of (A and B) Hey2, (C) COUP-TFII, (D) GLUT1 (E) GGTP and (F) Endoglin antibodies. (A) Hey2 is expressed in the endothelial cell lining (arrowhead) of AVM vessels. (B) Hey2 is expressed in micro vessels in control brain (arrowhead). Neuronal cells in control brain express Hey2 (arrow). (C) COUP-TFII is seen in the endothelial cell lining (arrowhead) of AVM vessels. (D) In control brain tissue GLUT1 expression is much less in the artery (arrowhead). (E) There is no expression of GGTP in control artery (arrowhead). (F) Endoglin is highly expressed in the microvessels in control brain (arrowhead). Magnification (A) and (C) 40X; (B and F) 10X and (D and E) 20X.
(TIF)

S3 Fig Immnuofluorescent staining with KLF2 antibody in AVM nidus. There is less expression of KLF2 in AVM vessels (green, arrow). Magnification 60X.
(TIF)
