# Supplementary figures and images for: Gene expression analysis of nidus of cerebral arteriovenous malformations reveals vascular structures with deficient differentiation and maturation
Source: PLoS One. 2018 Jun 13;13(6):e0198617. doi: 10.1371/journal.pone.0198617 (PMC5999265; doi:10.1371/journal.pone.0198617)

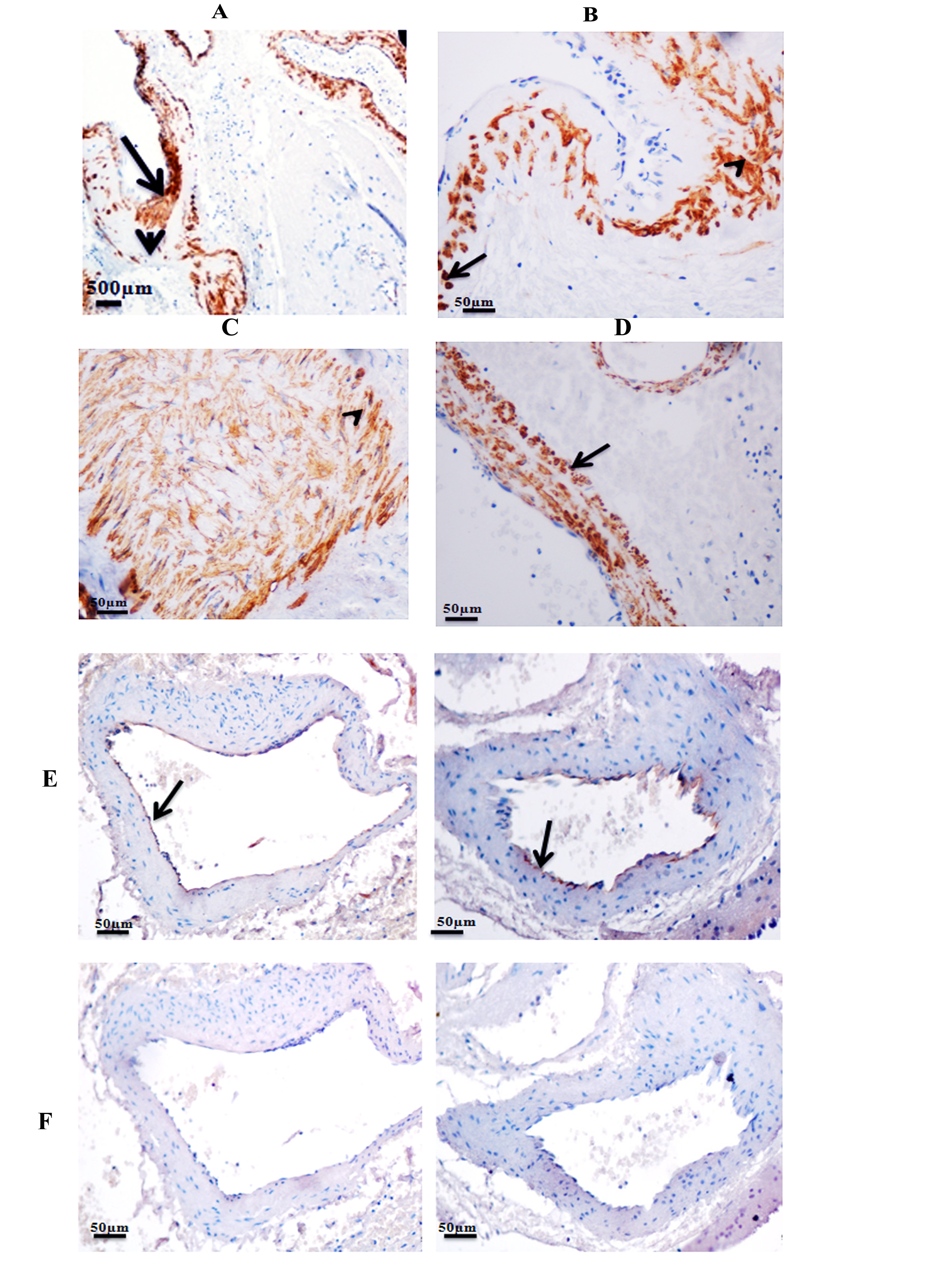

Supplement: S1 Fig — (A) α-SMA staining is seen in the endothelial cell lining of AVM nidus (arrow) and there is much less expression of α-SMA (arrowhead) in tunica media layer. 20X magnification. (B, C, D) 40X magnification images of α-SMA staining showing circular (arrow) and longitudinal smooth muscle cells (arrowhead) in AVM vessels. (E) PECAM-1 is expressed in the endothelial cell lining of AVM vessels. (B) Secondary isotype controls for PECAM-1 staining. 20X magnification. (TIF) [file pone.0198617.s001.tif]

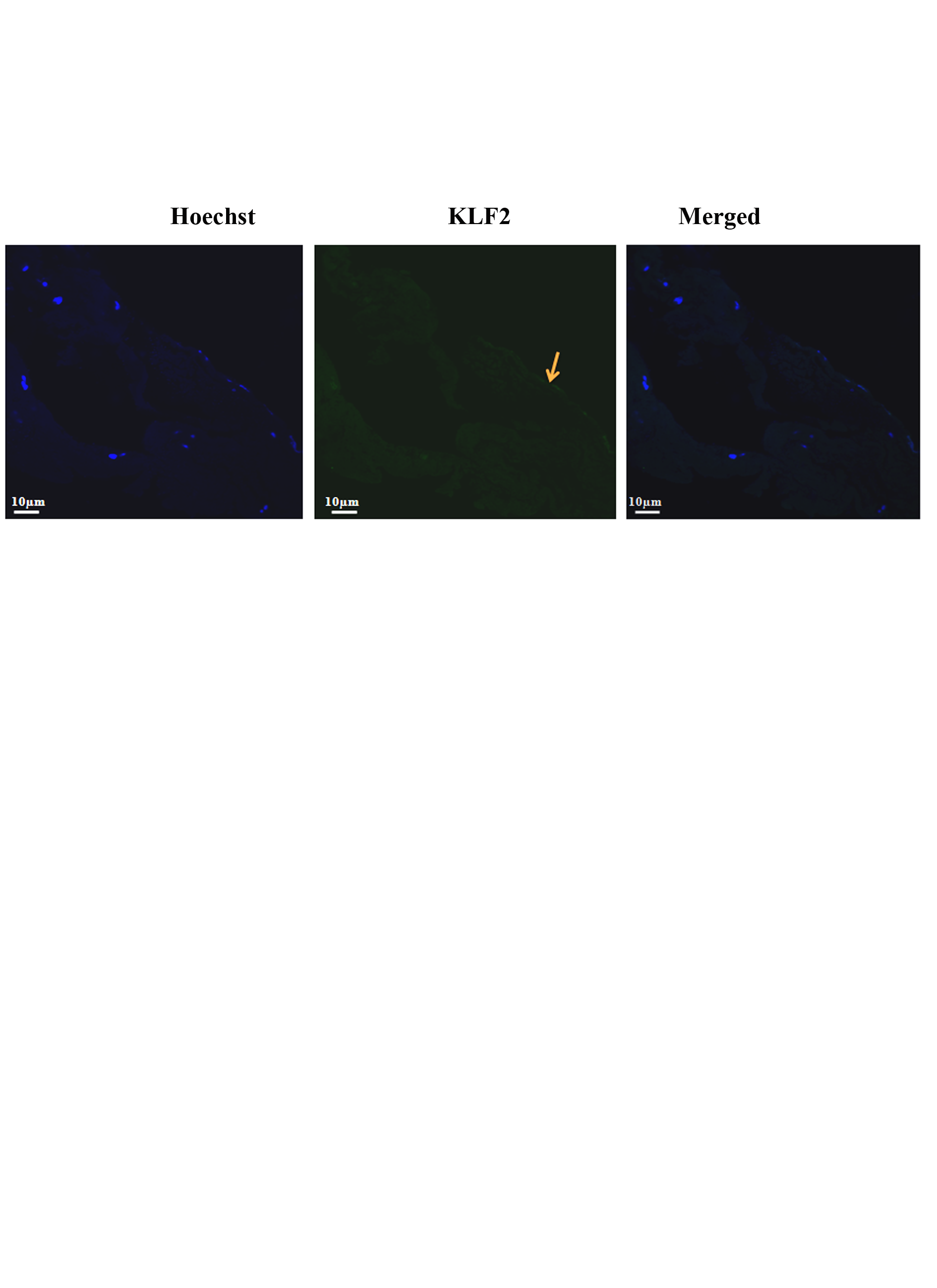

Supplement: S3 Fig — There is less expression of KLF2 in AVM vessels (green, arrow). Magnification 60X. (TIF) [file pone.0198617.s003.tif]
